# Chagas disease vector blood meal sources identified by protein mass spectrometry

**DOI:** 10.1371/journal.pone.0189647

**Published:** 2017-12-12

**Authors:** Judith I. Keller, Bryan A. Ballif, Riley M. St. Clair, James J. Vincent, M. Carlota Monroy, Lori Stevens

**Affiliations:** 1 Department of Biology, University of Vermont, Burlington, Vermont, United States of America; 2 Laboratorio de Entomología Aplicada y Parasitología, Escuela de Biología, Facultad de Ciencias Químicas y Farmacia, Universidad de San Carlos de Guatemala, Ciudad de Guatemala, Guatemala; Universite de Perpignan, FRANCE

## Abstract

Chagas disease is a complex vector borne parasitic disease involving blood feeding Triatominae (Hemiptera: Reduviidae) insects, also known as kissing bugs, and the vertebrates they feed on. This disease has tremendous impacts on millions of people and is a global health problem. The etiological agent of Chagas disease, *Trypanosoma cruzi* (Kinetoplastea: Trypanosomatida: Trypanosomatidae), is deposited on the mammalian host in the insect’s feces during a blood meal, and enters the host’s blood stream through mucous membranes or a break in the skin. Identifying the blood meal sources of triatomine vectors is critical in understanding Chagas disease transmission dynamics, can lead to identification of other vertebrates important in the transmission cycle, and aids management decisions. The latter is particularly important as there is little in the way of effective therapeutics for Chagas disease. Several techniques, mostly DNA-based, are available for blood meal identification. However, further methods are needed, particularly when sample conditions lead to low-quality DNA or to assess the risk of human cross-contamination. We demonstrate a proteomics-based approach, using liquid chromatography tandem mass spectrometry (LC-MS/MS) to identify host-specific hemoglobin peptides for blood meal identification in mouse blood control samples and apply LC-MS/MS for the first time to *Triatoma dimidiata* insect vectors, tracing blood sources to species. In contrast to most proteins, hemoglobin, stabilized by iron, is incredibly stable even being preserved through geologic time. We compared blood stored with and without an anticoagulant and examined field-collected insect specimens stored in suboptimal conditions such as at room temperature for long periods of time. To our knowledge, this is the first study using LC-MS/MS on field-collected arthropod disease vectors to identify blood meal composition, and where blood meal identification was confirmed with more traditional DNA-based methods. We also demonstrate the potential of synthetic peptide standards to estimate relative amounts of hemoglobin acquired when insects feed on multiple blood sources. These LC-MS/MS methods can contribute to developing Ecohealth control strategies for Chagas disease transmission and can be applied to other arthropod disease vectors.

## Introduction

Vector-borne diseases include some of the most complex disease systems, causing approximately 1.4 million deaths annually worldwide [1]. Chagas disease, a vector-borne neglected tropical disease is endemic in many parts of Latin America. It mostly occurs in communities with limited resources and traditional adobe or baroque houses made out of natural materials [1–3]. Chagas disease claims the lives of an estimated 12,500 people annually, with 8–10 million infected and over 60 million at risk of infection [4–6]. One-third of those infected with the Chagas parasite develop life-threatening illnesses, and it can take up to 20 years to develop diagnosable symptoms, making treatment difficult. Chagas disease is typically transmitted when a blood-feeding triatomine insect vector, also known as a kissing bug, deposits *Trypanosoma cruzi*-laden feces on the skin of a mammalian host. The parasite is subsequently introduced into the blood stream from the insect feces through a break in the skin or through mucous membranes. While congenital transmission occurs in 1–10% of infants born to Chagas-parasite positive mothers [4], *T*. *cruzi* transmission to humans occurs primarily during a blood meal from an infected triatomine vector [4, 7, 8].

Identifying blood meal sources of triatomine vectors is critical to understanding Chagas disease transmission dynamics and provides data for evidence-based vector control programs. There is currently no effective vaccine against Chagas disease and although two anti-Trypanosomal drugs, Nifurtimox and Benznidazole, are available, these have considerable side effects and are not always a solution to the overall disease management problem [2, 5, 9]. There are over 140 vector species across the Americas with varying degrees of importance regarding their roles in harboring and transmitting the Chagas parasite to humans [10]. Although the vectors are known to feed on reptiles, birds, and amphibians, the parasite can only reproduce in mammalian hosts, furthering the complexity of interrupting disease transmission [11, 12]. Understanding vector epidemiology and feeding prevalence can be an indicator of how well vector control strategies are working, aiding in Ecohealth control strategies where communities actively participate in reducing the conditions that can increase Chagas transmission [13–15]. Therefore, policies to adjust human behavior and to manage vectors remain more than a partner to medicinal therapeutics, and play increasingly important roles in controlling and preventing infection [14]. Critical to the development of effective policies is an accurate understanding of the sources of vector blood meals.

A complete understanding of the blood meal sources can be challenging for several reasons. DNA-based information is very powerful, relatively inexpensive and has been used in numerous studies to identify the species of blood meal sources. The relative stability of DNA compared to protein and the ability to PCR-amplify, sub-clone, and sequence DNA provides high sensitivity and specificity in blood meal identification, and therefore these methods are routinely used to determine blood meal sources from insect vectors [11–14, 16–26]. Although some methods can in part address issues such as DNA degradation [27, 28], most DNA-based methods work best with high quality DNA from recently fed vectors [22, 29–31]. Furthermore, given their reliance on an amplification step, DNA-based methods can lead to false positives from contaminating DNA that was not derived from the blood meal itself. Previously employed antibody-based techniques such as the precipitin and antisera tests require fresh material stored at cold temperatures and specific antibodies of possible host species in an area [32–38].

Therefore, additional tools for identification and quantification of insect vector blood meal sources are desirable. Here we demonstrate a liquid chromatography-tandem mass spectrometry (LC-MS/MS) approach based on the identification of highly stable hemoglobin proteins [39–41] which are some of the most abundant proteins in any blood meal [42]. Indeed, the remarkable stability of iron-bound hemoglobin is illustrated by the high amounts of iron and porphyrins derived from hemoglobin using time-of-flight secondary ion mass spectrometry that have been detected in a 46-million-year old fossilized mosquito [39]. In addition, hemoglobin has been detected up to 309 days post-molting under laboratory conditions in ticks through hemoglobin sequence searching [41]. Our approach, based on publicly available DNA and protein sequences, can be highly precise when using the entirety of published sequences and does not require creating spectral libraries of potential vertebrate hosts as required for other mass spectrometry based techniques that use spectral matching [30]. Because of the high precision, mass spectrometry can be used to accurately identify hemoglobin peptide sequences, many of which are unique to specific vertebrate classes, orders, families, genera, and even species [40–42].

The purpose of this study was to determine if we can apply a proteomic-based, LC-MS/MS approach for identification of blood meal sources of Chagas disease insect vectors. For this purpose, we first used blood from *Mus musculus* (house mouse) to validate our ability to identify mouse hemoglobin peptides from LC-MS/MS to predictions based on a database of hemoglobin sequences we curated through GenBank of the National Center for Biotechnology Information (NCBI) [43]. Subsequently, we provide the first identification of vertebrate host hemoglobin peptides in Chagas disease vectors to determine blood meal sources, using the same LC-MS/MS approach applied to the mouse blood controls. This is the first application of LC-MS/MS for blood meal identification from field-collected, arthropod disease vectors, verified with DNA-based analysis. We confirm our blood meal species identification with DNA analysis based on sequencing the mitochondrial 12S and 16S ribosomal genes and specifically examine insect vectors that have proved challenging for DNA analysis (i.e., stored in ethanol and collected before or after the insect had died). Finally, we demonstrate the potential to estimate absolute quantities of hemoglobin leading the way for further studies quantifying amounts of hemoglobin from arthropod disease vectors that have fed on multiple taxa.

## Methods

**Fig 1:**
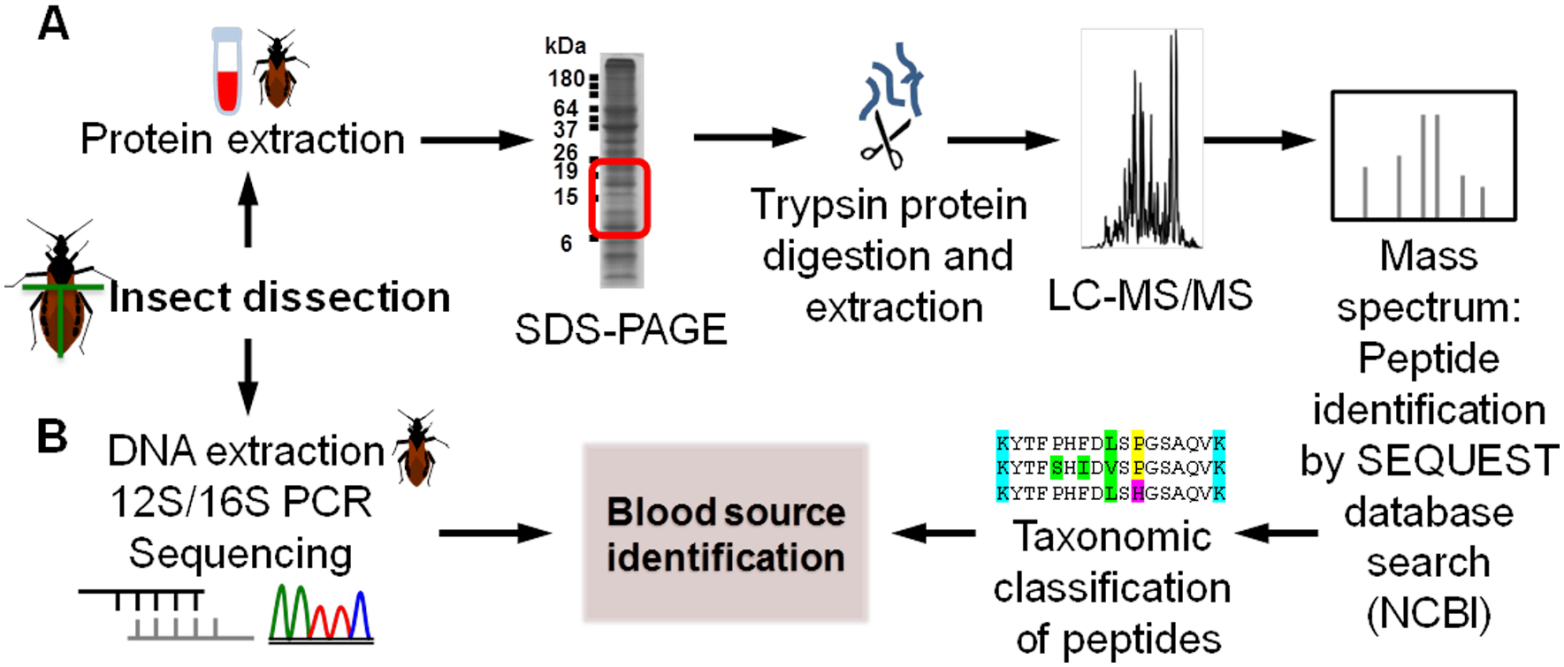
General workflow describing LC-MS/MS-based and DNA-based identification of blood meal sources. Insects were dissected into left and right abdomen, and hemoglobin peptides from (A) mouse blood as well as triatomine insect vectors were identified with LC-MS/MS, while (B) triatomine insect vector blood meals were also identified with DNA-based methods for comparison.

The general workflow to identify blood meal sources is shown in Fig 1. We describe that workflow below.

### Ethics statement

All procedures were reviewed and approved by the Institutional Animal Care and Use Committee (IACUC) at the University of Vermont in accordance with the requirements of the Office of Laboratory Animal Welfare (IACUC protocol 12–045). Mouse blood samples for this study were obtained and treated in accordance with an IACUC-approved protocol encouraging “tissue sharing” of post-mortem tissue.

### Collection and storage of mouse blood controls

In order to evaluate LC-MS/MS for blood source identification using hemoglobin peptides, we first validated our ability to identify *M*. *musculus* blood. Whole blood samples were drawn from three two-month-old Dicer mice [44, 45] immediately following euthanization by cervical dislocation. These transgenic mice with different genetic backgrounds were derived from the cross of C57 and FVB mice [44, 45]. To investigate the role of the anticoagulant ethylenediamine tetraacetic acid (EDTA), blood samples were placed in one of three storage conditions: (1) EDTA (100 mM EDTA; Sigma-Aldrich, Saint Louis, Missouri, USA), (2) dipotassium EDTA (18 mM K_2_EDTA; BD Vacutainer, Becton, Dickinson and Company, Franklin Lakes, New Jersey, USA), and (3) no preservative. Immediately following collection, blood tubes were inverted several times to prevent clotting in the EDTA-containing tubes and then stored at -20°C for 2 months at which time blood proteins were extracted as described below.

### Collection and dissection of insect vectors

Four *Triatoma dimidiata* (Hemiptera: Reduviidae) insect vectors were collected from houses in villages in El Salvador between September and November 2012 (Table 1). Three of these specimens were alive and one was dead at the time of collection (Table 1). Collected insects were stored in 95% ethanol, 5% glycerol, and kept at room temperature for 1–3 months before being stored at -20°C until dissected in January of 2014. The insect abdomen (consisting of the caudal end of the insect posterior to the crop) was separated into the left and right halves. Specimen halves were randomly assigned to protein or DNA analysis.

**Table 1:** *T*. *dimidiata* insect vectors collected from houses in villages in El Salvador, abdomen halves assigned to LC-MS/MS protein or DNA analysis.

Sample	Analysis	Sex	Collection Location			Collection
			Village	Subvillage	Ecotope	
FER 0051–1	LC-MS/MS	M	Azacualpa	El Tablón	domestic	alive
FER 0051–2	DNA					
FER 0076–1	DNA	M	Azacualpa	Ocotillo	domestic	dead
FER 0076–2	LC-MS/MS					
FER 0101–1	DNA	M	Cañaverales	Dolores	peridomestic	alive
FER 0101–2	LC-MS/MS					
FER 0112–1	LC-MS/MS	F	Cañaverales	Plantanares	peridomestic	alive
FER 0112–2	DNA					

1, left abdomen; 2, right abdomen; M, male; F, female.

### Protein extraction, SDS-PAGE, and mass spectrometry

We extracted proteins from *M*. *musculus* blood by adding 60 μl of mouse blood from the storage conditions listed above, to 20 μl of 95°C 4X denaturing sampling buffer to a final concentration of 5% bromphenol blue, 150 mM Tris pH 6.8, 2% SDS, 5% β-mercaptoethanol, 7.8% glycerol, and heating for 5 min at 95°C. Each sample was serially diluted with 1X denaturing sampling buffer such that the equivalent of 2 μl of mouse blood was run and analyzed per gel lane. For insect samples, proteins were extracted by adding 100 μl of 95°C denaturing sampling buffer (see above) per 0.1 g of insect tissue and ground with a clean glass rod in a fume hood. The samples were subsequently heated for 5 min at 95°C.

Each mouse blood and insect sample was then centrifuged at 16,000 X g for five minutes, and 20 μl of the supernatant was subjected to denaturing 15% SDS-PAGE (37.5 acrylamide: 1 bis-acrylamide) and stained with Coomassie blue. Gel regions surrounding the molecular weight of hemoglobin were excised and diced into 1 mm cubes. In-gel digestion with trypsin, peptide extraction and LC-MS/MS analysis using a linear ion trap-orbitrap (LTQ-Orbitrap; Thermo Electron, Waltham, Massachusetts, USA) was performed as described previously [46] except that all spectra were acquired in the orbitrap for the mouse blood and field-collected insect vector samples.

### Using mass spectrometry for the quantification of total hemoglobin and taxon-specific hemoglobin

In some cases, Chagas vectors take blood meals from multiple sources [12]. Therefore, methods that could apportion the contribution of distinct taxa to the total blood meal would be desirable. Successfully accomplishing this using common DNA and mass spectrometry approaches are strongly dependent on well-curated sequence databases. In the case of DNA, taxon-specific PCR primers may be used for targeted amplification analyses to capture qualitative (agarose gel stained PCR products) or quantitative (qPCR) information about the contribution of particular taxa to a blood meal. Additionally, PCR primers specific to regions that are constant across taxa could be used to amplify across a variable region that would then require cloning or Next-generation sequencing of PCR products in order to attribute the percent any one taxon contributed to the total.

Determining the sequence of amino acids within a peptide by mass spectrometry is predominantly accomplished by matching observed tandem mass spectra with theoretical tandem mass spectra calculated from known protein sequences. While the canonical hemoglobin sequences for most organisms on which Chagas vectors feed are known, it remains a formal possibility that some hemoglobin sequences are not present in current databases; however we based our analysis on hemoglobin because of the stability of the molecule as demonstrated by the ability to identify it over geologic time [39]. Furthermore, it would be desirable to ascertain the contributions of each blood source present in the vector at a given time. A proteomics method to quantify the absolute amount of hemoglobin present in a kissing bug, and the percentage of hemoglobin from any known or unknown species, could take advantage of the fact that some regions of hemoglobins are highly polymorphic while other regions are invariant [41, 47]. The method would involve quantifying the amount of an invariant peptide to determine the total amount of hemoglobin ingested and also quantifying species-specific peptides to ascertain the relative contribution of a given species to the total. If the kissing bug harbored blood from an unknown species then the sum of the species-specific peptides would be less than the amount of the invariant peptide. In proteomics this approach employs stable isotope-containing peptide standards and is known as Absolute QUAntification or AQUA [48].

Quantification using a stable isotope-containing standard peptide was done as described previously [49] using the synthetic AQUA peptide LLVVYPWTQR which contained ^13^C_5_, ^15^N_1_-proline synthesized at Cell Signaling Technology (Danvers, Massachusetts, USA). LC-MS/MS methods were as described above, except that only MS1 spectra were acquired in the orbitrap for quantification, while MS2 spectra were collected in the linear ion trap mass spectrometer (LTQ)

### Peptide and blood meal identification

LC-MS/MS does not directly sequence the amino acids in a peptide, but gives a spectrum of the masses associated with fragmenting a peptide which can then be matched with high accuracy to expected peptide sequences in an underlying database [50]. Mass spectra were searched using SEQUEST (Thermo Electron V26.12) against a custom forward and reverse concatenated database using a target-decoy approach [51] and allowing for variable oxidation of methionine (+15.9943 Da) and acrylamidation of cysteine (+71.0371 Da). The custom database contained vertebrate hemoglobin sequences (available data for amphibians, reptiles, birds, and mammals) extracted from GenBank on 20 January 2016 with “hemoglobin” in any curated field [43]. We used this strong comparative database with over 17,000 entries to identify hemoglobin peptides in a given sample.

Only doubly- and triply-charged peptide ions were considered. Peptide filtering criteria were: (1) XCorr values greater than or equal to 2.5 (*z* = 2) or 3 (*z* = 3); (2) measured precursor masses +/- 5 PPM; (3) unique ΔCn values greater than or equal to 0.1; and (4) no missed tryptic cleavages except at the extreme N- or C-termini of peptides where more than one R or K in a row was allowed. These stringent filters resulted in no reverse database matches and thus gave peptide false discovery rates of less than 0.01%. In order to identify the vertebrate species to which the amino acid peptide sequences matched, protein entries from the database were subjected to *in silico* tryptic cleavage using the Pyteomics [52] python tools and library. Taxonomic lineage for each protein was collected from the NCBI taxonomy database [53] (20 January 2016). Peptides were associated with the parent protein taxonomic lineage and stored in a relational database for easy retrieval, allowing us to determine to which vertebrate species an identified hemoglobin peptide matched.

Hemoglobin proteins are sufficiently conserved across these vertebrate taxa to allow mapping of individual peptides to the alpha and beta chains. Peptides are identified with the chain (alpha or beta) and first and last amino acid (i.e. alpha_17–31, or beta_0–8) as mapped to GenBank NP_032244.2, BAG16710.1 for mouse, and P07405.1, XP_003992931.2 and P60529.1, P60524.1 for cat and dog, respectively. However, some variation occurs due to polymorphisms in arginine and lysine residues where the trypsin cuts occur. Among the spectra for a sample, there can be more than one that represent the same peptide. We use the term spectral counts to refer to the number of times a peptides was identified and peptide variant to refer to peptides that map to the same position but vary in a few amino acids. Some hemoglobin peptides are polymorphic within species, others are unique to a particular species, whereas others may be found in multiple species. In addition, some alpha or beta hemoglobin sequences have a variable methionine at the extreme amino-terminal, which corresponds to alpha_0 or beta_0. If the methionine is absent, the sequence starts with alpha_1 or beta_1 with our designation.

**Fig 2:**
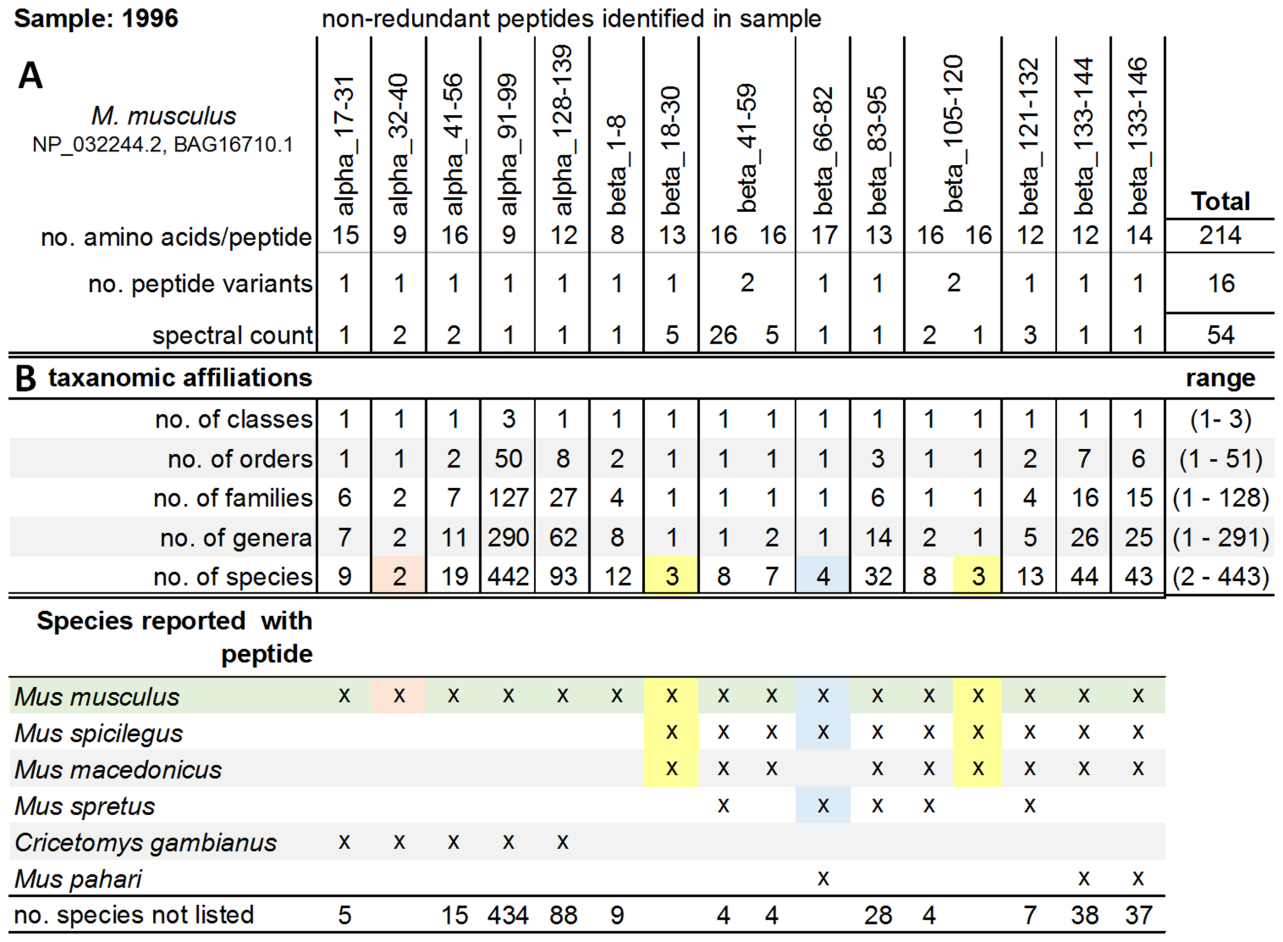
Pipeline for inferring blood meal sources from hemoglobin peptides illustrated for mouse blood sample 1996. (A) Peptides identified by SEQUEST mapped to alpha and beta hemoglobin, using GenBank NP_032244.2, BAG16710.1 reference alignments for *M*. *musculus* hemoglobin (amino acid 0–141 for alpha and 0–146 for beta hemoglobin). Peptides are named based on hemoglobin chain and the position of the first and last amino acid (i.e. alpha_17–31). When more than one peptide mapped to a position (e.g., beta_41–59), corresponding data for both peptide variants are shown. (B) Taxonomic affiliations for each peptide that matched five species or less are tabulated, in this case peptide alpha_32–40 matched two species (orange highlight), two peptides matched three species (yellow), and one peptides matched four species (blue). The peptides are then examined to determine if they are consistent with each possible species. ‘x’ represents a peptide that matched the particular species in question.

Because some peptides have been reported in multiple species and some polymorphisms may not appear in the curated database, we developed a pipeline (Fig 2) to infer the most likely blood source and verified it with the mouse blood samples. Subsequently we applied the same pipeline for identifying blood meal sources of triatomine insect vectors. The pipeline tabulates the potential taxa represented by the peptides identified by LC-MS/MS in a sample. For identifying the potential blood source of a given sample, we calculated two summary statistics, the first quantifying the protein coverage of a species’ hemoglobin by peptide identified in a given sample. The second, based on the spectral count, was the percentage LC-MS/MS spectra of a given sample matching to the hemoglobin peptides identified of a particular species. The taxonomic identification was based on the best match, where the highest matches of the two summary statistics indicate the most likely blood source for each sample (see Fig 2 and Table 2 for more detail).

**Table 2:** Using the information from the pipeline (Fig 2), two summary statistics quantify the strength of blood meal source identification.

Species reported with peptide	Total peptide matches per taxon	Total peptide non-matches per taxon	Percent peptides identified matching	Percent spectral count matching
*Mus musculus*	16	0	100.0%	100.0%
*Mus spicilegus*	10	6	62.5%	85.2%
*Mus macedonicus*	9	7	56.3%	83.3%
*Mus spretus*	5	11	31.3%	61.1%
*Cricetomys gambianus*	5	11	31.3%	13.0%
*Mus pahari*	3	13	18.8%	5.6%

The percentage of peptides identified quantify whether a particular peptide does/does not match the proposed blood source. The percent of spectral counts matching a particular taxon provides a semi-quantitative measure of relative abundance. The species with the highest summary statistics is determined to be the most likely blood source, in this case *M*. *musculus* (green highlight).

### DNA extraction and 12S/16S mitochondrial sequencing of insect abdomens

We verified our results based on LC-MS/MS with a more traditional blood meal identification method for our insect vector samples. From *Triatoma dimidiata* insect abdomen halves, DNA was extracted using the DNeasy Blood and Tissue Kit (Qiagen, Valencia, CA) as previously described [19, 54, 55] and identification of blood meal sources was similar to previously published methods based on PCR amplification and sequencing of vertebrate mitochondrial genes [20, 54] except that multiple sets of primers were used and PCR products were directly sequenced, omitting the cloning step. Briefly, the PCR reaction used primers specific for vertebrate mitochondrial DNA coding for fragments of the 12S and 16S ribosomal RNA gene (hereafter referred to as 12S or 16S primers). Because primer efficiency depends on the blood meal source, three sets of vertebrate 12S and one set of vertebrate 16S primers were used [55–57]. A positive control and negative control (PCR-grade water) were included in each set of PCR amplifications. An ethidium bromide stained, 1.5% agarose gel was used to verify the 215 bp (Kitano), ~100 bp (Melton), 98 bp (Karlsson 16 S), or 111 bp (Karlsson 12 S) PCR products, which were sequenced using BigDye v3.1 (Applied Biosystems, Foster City, CA, USA) and subsequently analyzed with an ABI PRISM 3730xl DNA analyzer (Beckman Coulter, Fullerton, CA, USA). Sequence alignments and editing were done with Sequencher v4.10 (Gene Codes Corporation, Ann Arbor, MI, USA). Taxonomic identification of the sequences was based on the best match of 215 bp (Kitano), ~100 bp (Melton), 98 bp (Karlsson 16 S), or 111 bp (Karlsson 12 S) using the NCBI BLAST algorithm [58].

## Results

In this study, we show that a proteomics-based approach using LC-MS/MS can be a valuable additional technique for identifying blood meal sources using mouse blood to verify the approach as well as identifying blood meals from Chagas disease triatomine insect vectors. First, we show LC-MS/MS is able to unambiguously identify peptides. We then show how these peptide data, combined with our analysis pipeline, allowed us to correctly identify hemoglobin peptides from *M*. *musculus* blood to correct animal species with strong support. Finally, we were able to identify dog and cat as blood meal sources from field collected *Triatoma dimidiata* and employ a LC-MS/MS-based method for quantifying a blood meal source.

### Mouse blood

**Fig 3:**
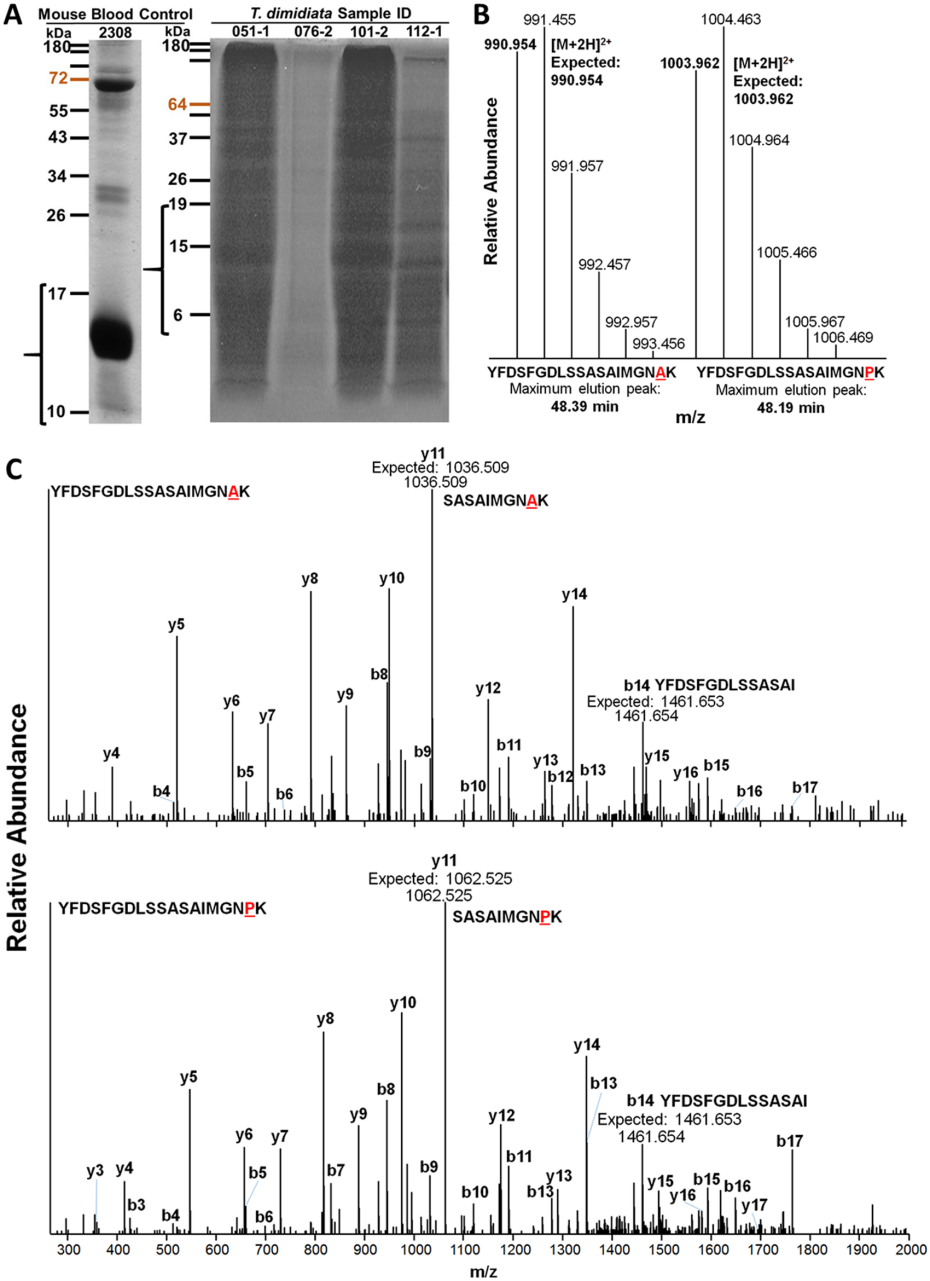
Proteomics-based, LC-MS/MS distinguishes unique but nearly identical peptides. (A) Representative SDS-PAGE results of mouse blood samples and of four *T*. *dimidiata* insect vectors. (B) MS1 spectra of two doubly-charged peptide ions differing by a single amino acid- YFDSFGDLSSASAIMGNAK and YFDSFGDLSSASAIMGNPK, identified in mouse blood control sample 2308. Mass to charge ratios for these peptides differ only by the difference between the variable amino acid (alanine or proline). The two peptides also elute at slightly different times. (C) Low energy collision-induced dissociation fragmentation (MS2) mass spectra of the aforementioned peptide ions allow determination of the peptide sequence. Peaks are labelled as per convention with b-type fragment ions (those derived from the amino terminus) and y-type fragment ions derived from the carboxyl-terminus. Given the variable amino acid is the penultimate carboxyl-terminal amino acid, y2 and higher y-type ions differ by the mass variability between alanine and proline (e.g. y11), while almost all b-type ions (e.g. b14) show equal *m/z* measurements. Expected and observed masses for identified fragment ions can be found in S1 and S2 Tables.

We were able to identify hemoglobin protein peptides unambiguously with our proteomics-based, LC-MS/MS approach (Fig 3, Table 3). Peptides differing only by a single amino acid, such as YFDSFGDLSSASAIMGNAK and YFDSFGDLSSASAIMGNPK, could be identified with high confidence in our mouse blood (Fig 3). As the mouse blood used in these experiments came from non-isogenic strains of mice with hybrid genetic backgrounds, we could often identify peptide variants for a single peptide position.

**Table 3:** Tryptic peptides identified in mouse blood control samples, including hemoglobin position and spectral counts.

Peptide	Hemoglobin position	Mouse blood control sample		
		1996	2308	2310
		Spectral Counts		
IGGHGAEYGAEALER	alpha_17–31	1	2	1
VGSHAGEYGAEALER ^a^	alpha_17–31		1	
MFASFPTTK	alpha_32–40	2	1	
TYFPHFDVSHGSAQVK	alpha_41–56	2	1	
VADALANAAGHLDDLPGALSALSDLHAHK	alpha_62–90			5
LRVDPVNFK	alpha_91–99	1		
FLASVSTVLTSK	alpha_128–139	1		
VHLTDAEK	beta_1–8	1		
AAVSGLWGK	beta_9–17			6
NVADEVGGEALGR ^a^	beta_18–30		11	11
VNSDEVGGEALGR	beta_18–30	5	3	
YFDSFGDLSSASAIMGNAK	beta_41–59	26	24	27
YFDSFGDLSSASAIMGNPK	beta_41–59	5	3	
KVITAFNDGLNHLDSLK	beta_66–82	1	2	
VITAFNEGLK	beta_67–76		1	
VITAFNDGLNHLDSLK	beta_67–82		5	
GTFASLSELHCDK	beta_83–95	1		
LHVDPENFR	beta_96–104		1	
LLGNAIVIVLGHHLGK	beta_105–120	1		
LLGNMIVIVLGHHLGK	beta_105–120	2	1	
DFTPAAQAAFQK	beta_121–132	3		1
VVAGVAAALAHK	beta_133–144			4
VVAGVATALAHK	beta_133–144	1		
VVAGVATALAHKYH	beta_133–146	1		
No. of non-redundant peptides		16	13	7
No. of peptide positions	16	13	8	7
No. of spectral counts		54	56	55

^a^ indicates peptides not matching the known blood sources, *M*. *musculus*; 1996, 100 mM EDTA; 2308, 18 mM K_2_EDTA; 2310, no preservative

Our novel LC-MS/MS method allowed us to correctly identify the taxon of origin when analyzing hemoglobin peptides isolated from *M*. *musculus* (Table 3). We identified 24 non-redundant hemoglobin protein peptides from the 165 mass spectra collected in the three mouse blood samples. None of the 24 peptides was unique to *M*. *musculus*, however 22 of the 24 peptides (91.6%) and 153 of the 165 spectral counts (92.7%) matched previously reported *M*. *musculus* hemoglobin sequences in GenBank, making it the most supported blood source species (Fig 2 and S1–S3 Figs). The 22 peptides matching records of *M*. *musculus* in GenBank matched anywhere from two to as many as 442 taxa. Of the two peptides (0–2 per sample) where the top match was not *M*. *musculus*, one occurred at the alpha_17–31 position and the other at beta_18–30. However, for these two peptides, the number of amino acids matching *M*. *musculus* as the blood source was high (Table 4).

**Table 4:** Summary statistics and blood source identifications of mouse blood controls and triatomine insect samples.

Sample	Blood source identification	Percent peptides identified matching	Percent spectral count matching	Percent amino acids identified matching
**Mouse blood samples**				
1996	*M*. *musculus*	100.0%	100.0%	100.0%
2308	*M*. *musculus*	84.6%	78.57%	96%
2310	*M*. *musculus*	85.7%	80.0%	98%
***T*. *dimidiata* insect samples**				
051	*F*. *catus*	85.7%	92.3%	99.5%
076	*C*. *lupus familiaris*, *C*. *latrans*, or *C*. *brachyurus*	100.0%	100.0%	100%
101	*C*. *lupus*	92.3%	95.7%	99.5%
112	*C*. *lupus*	100.0%	100.0%	100%

1996, 100 mM EDTA; 2308, 18 mM K_2_EDTA; 2310, no preservative

**Fig 4:**
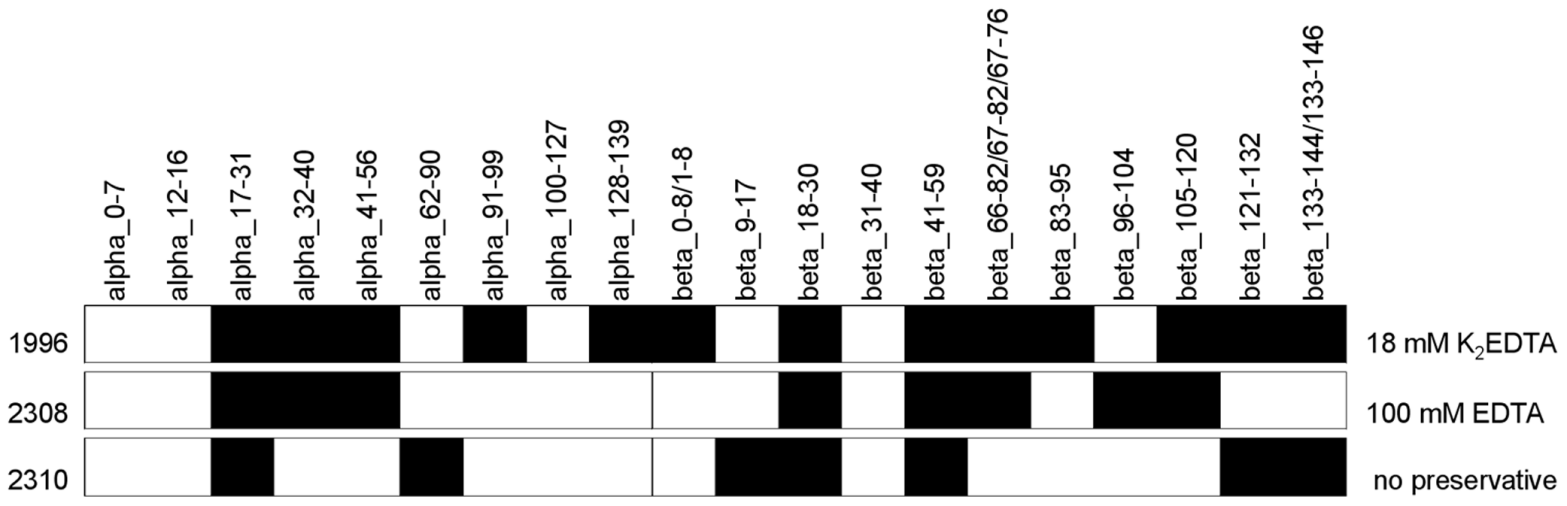
Hemoglobin protein peptide coverage from identified tryptic peptides for three mouse blood samples. Three mouse blood samples stored in varying amounts of EDTA are shown. The peptides are shown with equal width, not in proportion to the length of the peptide, and only peptides greater than four amino acids are shown.

In terms of protein coverage, we found 16 of the 20 (80%) expected tryptic peptides (greater than 4 amino acids, Fig 4). Trypsin digestion is expected to yield 9 alpha and 11 beta hemoglobin peptides greater than 4 amino acids in length from *M*. *musculus* hemoglobin based on GenBank entries NP_032244 and BAG16710. Of the 16 peptides that we identified, one (alpha_17–31) had two peptide variants (Table 3). When blood was stored in EDTA (samples 1996, 2308), more non-redundant peptides were identified, while spectral counts were similar in all three treatments (Table 3).

### Identification of blood meal sources of triatomine insect vectors

Our LC-MS/MS method identified blood meals from insects collected alive and dead; however, blood meals were not detected with DNA sequencing methods for three of the four samples. By LC-MS/MS, Canidae species, especially dog, was the most strongly supported blood meal source in three (076, 101, 112) of the field collected samples and cat in the other (051) (Table 4 and S4–S7 Figs). These results are based on 22 non-redundant hemoglobin peptides although the number of non-redundant peptides per samples was 50% fewer (8 for the blood meal from the insect compared to 12 for mouse blood) and the number of spectra per sample was at most half of the mouse blood (3–22 for the blood meal from the insect compared to 54–56 for mouse blood) (Table 5). Two of the 7 peptides from sample 051 were unique to cat, and 6 of the 7 (85.7%) matched cat hemoglobin sequences in GenBank (S4 Fig). The next closest match was to two members in the squirrel (Sciuridae) family (14%). None of remaining peptides from the other three samples were unique to dog, and all except one were previously reported in dog (86.2–100% peptides matching, Table 4). Spectral count matching to the top identified species was also high across the four samples (92.3–100%, Table 4). The next closest matches were *Canis latrans*, and *Chrysocyon*. *brachyurus*, both members of the Canidae family. In terms of amino acids identified, >99% of amino acids identified matched the most likely blood source(s) (Table 4).

**Table 5:** Peptides identified in triatomine insects, including hemoglobin position and spectral counts.

Peptide	Hemoglobin position	*T*. *dimidiata* samples			
		FER051	FER076	FER101	FER112
		Spectral Counts			
IGGHAGDYGGEALDR	alpha_17–31			1	1
TFQSFPTTK	alpha_32–40			1	2
TYFPHFDLSHGSAQVK	alpha_41–56	1			
TYFPHFDLSPGSAQVK	alpha_41–56			3	
KVADALTTAVAHLDDLPGALSALSDLHAYK	alpha_61–90			1	
VADALTQAVAHMDDLPTAMSALSDLHAYK	alpha_62–90	4			
VADALTTAVAHLDDLPGALSALSDLHAYK	alpha_62–90			2	2
VADALTTAVSHIDDLPGALSALSDLHAYK ^a^	alpha_62–90			1	
LRVDPVNFK	alpha_91–99			1	
FFAAVSTVLTSK	alpha_128–139			3	1
VHLTAEEK	beta_1–8			3	
SLVSGLWGK	beta_9–17		1		
VNVDEVGGEALGR	beta_18–30	1	1	1	
LLIVYPWTQR	beta_31–40		1		1
FFDSFGDLSTPDAVMSNAK	beta_41–59			3	3
FFQSFGDLSSADAIMSNSK	beta_41–59	3			
KVLDSFSDGLK ^a^	beta_66–76	1			
VLNSFSDGLK	beta_67–76	2			1
LHVDPENFK	beta_96–104			1	
VVAGVANALAHK	beta_133–144			1	1
VVAGVASALAHR ^b^	beta_133–144	1			
No. of non-redundant peptides		7	3	14	8
No. of peptide positions	16	6	3	12	8
No. of spectral counts		13	3	22	12

^a^ indicates peptides not matching the most likely blood meal (051, cat; 076, 101, 112, dog).

^b^ indicates a peptide that was added to GenBank 29 Dec 2016, after the original database was created.

**Fig 5:**
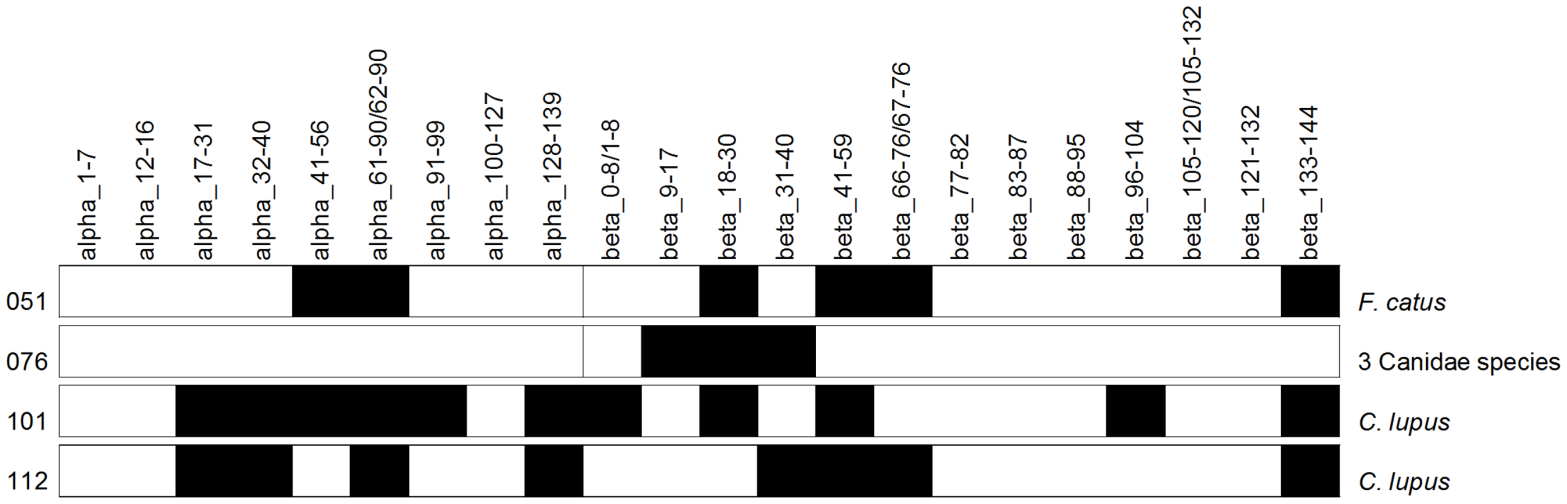
Hemoglobin protein peptide coverage from identified tryptic peptides from blood meals of four triatomine insects. Triatomine insect vectors were collected alive (051, 101, 112) and dead (076). The peptides are shown with equal width, not in proportion to the length of the peptide, and only peptides greater than four amino acids are shown.

Although the match to previously reported hemoglobin sequences in GenBank was high, the number of non-redundant peptides and the peptide coverage was low (range 3–14 peptides with 13.6% - 28% coverage, Fig 5), demonstrating that only a few peptides are sufficient to identify a blood meal. Note that for cat there are 10 alpha and 12 beta predicted hemoglobin tryptic peptides and for dog there are 9 alpha and 13 beta predicted tryptic peptides, considering peptides greater than 4 amino acids. Of the 22 peptides identified in triatomine insect vectors, only two peptides were variable within species, alpha_62–90 in sample 101, and beta_66–76 in sample 051. Of the insect vectors collected alive (051, 101, 112), more non-redundant peptides as well as spectral counts were identified (Table 5). Taxonomic resolution was lower for the sample collected dead, only 3 peptides were identified. Still, the blood meal source could be resolved to three possible species (*C*. *lupus familiaris*, *C*. *latrans*, *C*. *brachyurus*) in the Canidae family.

While LC-MS/MS was able to identify blood meals in all four insects, blood meals were not identified with more traditional 12S/16S mitochondrial DNA sequencing methods for three of the four samples because there was no visible band from the PCR reaction, while positive controls amplified successfully. Our previous studies demonstrated lack of PCR product indicates no recent blood meal rather than PCR inhibition [13, 22] and experimental studies have revealed that DNA detection can drop off as early as 1–2 weeks after a blood meal depending on blood meal host species [59]. The DNA sequence and LC-MS/MS results did agree for the one insect where they were both available (Table 6). Sample FER112-2 had a visible PCR product for 3 primer sets (the 12S Karlsson primers did not amplify), and matched *C*. *lupus familiaris* (domestic dog) 100%, 100% and 99% for 12S Kitano, 16S Karlsson and 12S Melton primers, respectively.

**Table 6:** Comparison of LC-MS/MS-based and DNA-based blood meal source identification methods.

Sample	12S/16S blood meal identification	LC-MS/MS blood meal identification
FER 051	None	*F*. *catus*
FER 076	None	3 Canidae species
FER 101	None	*C*. *lupus familiaris*
FER 112	*C*. *lupus familiaris*	*C*. *lupus familiaris*

### Quantification of blood meal sources of triatomine insect vectors

**Fig 6:**
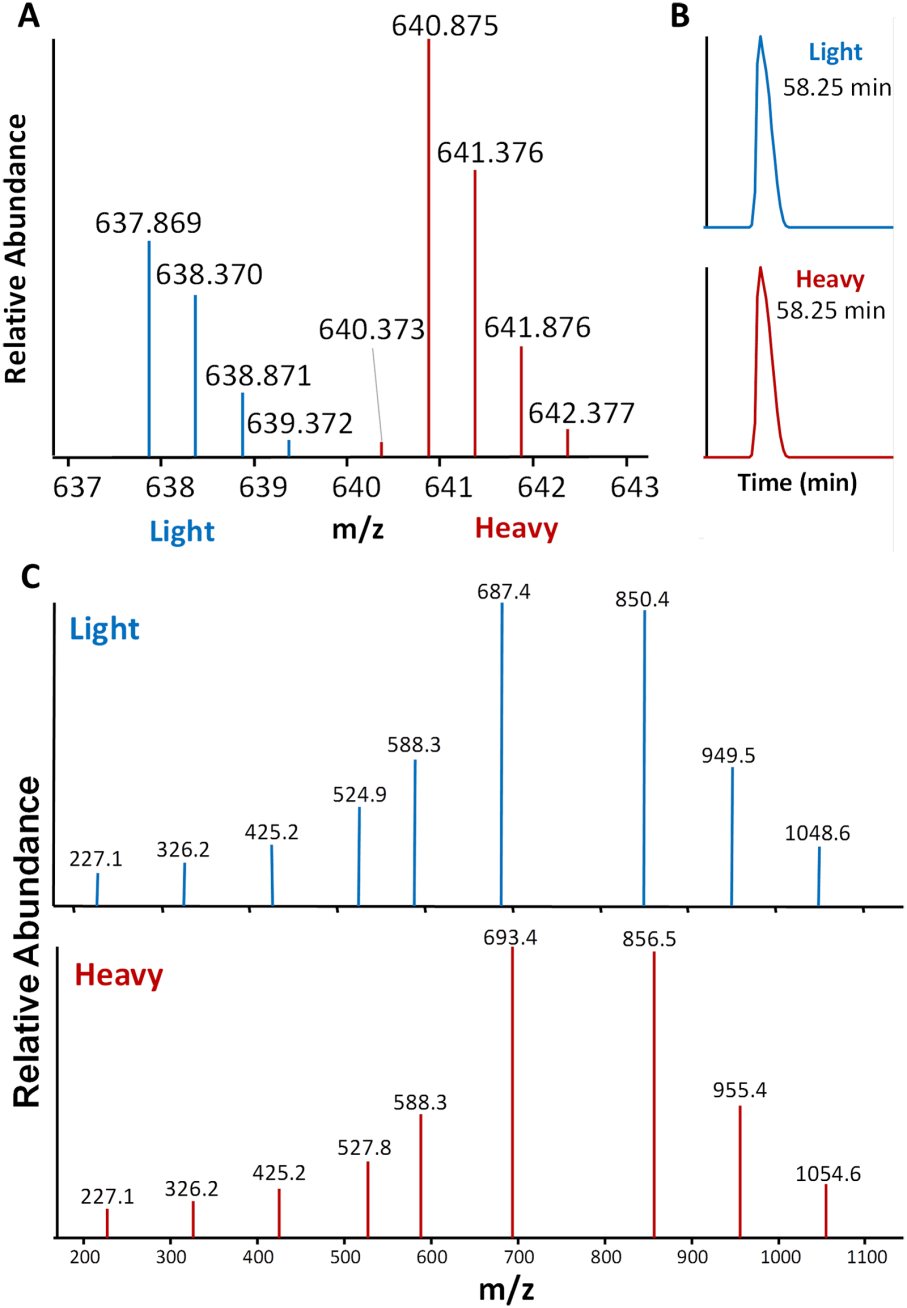
Stable isotope-labeled standard facilitates quantification of native hemoglobin peptide. (A) MS1 spectrum showing the relative abundance of a native hemoglobin peptide derived from the blood meal of a field-collected kissing bug (blue isotopic envelope) in comparison to an introduced synthetic peptide standard (red isotopic envelope) of the same sequence containing a ^13^C_5_, ^15^N_1_-labeled proline. (B) Partial chromatogram showing the co-elution of the monoisotopic masses of both the native “light” and synthetic “heavy” peptide ions shown in A. (C) Low-energy collision-induced dissociation MS2 fragmentation spectra of the doubly-charged precursor ions for the native and synthetic peptides.

As a first-step in developing a method for quantifying blood meals, we demonstrated the quantification of a highly-conserved mammalian hemoglobin peptide arising from the blood meal of a field-collected kissing bug. To tryptic peptides derived from the insect blood meal was added a highly conserved synthetic tryptic peptide from beta-hemoglobin, LLVVYPWTQR, which harbored a ^13^C_5_, ^15^N_1_-labeled proline residue (ΔM = 6.012). As expected, both the insect blood meal-derived peptide and the synthetic standard eluted at the same time enabling a direct comparison of the relative abundance of each in precursor (MS1) scans. Furthermore, the MS2 scans of the native blood meal and synthetic peptides unambiguously distinguish the two peptides as their spectra differ only in peptide fragments containing the heavy-labeled peptide (Fig 6). In this example, the relative abundance of the native peptide is approximately 50% of the synthetic peptide; the heavy-to-light ratio of synthetic to native peptide was 1.94:1 (4.55x10^7^:2.35x10^7^ comparing monoisotopic peak intensities).

## Discussion

We demonstrate the application of LC-MS/MS for the identification of blood sources based on hemoglobin peptides. After validating our methodology with mouse blood control samples, we identified blood meal sources of Chagas disease insect vectors. This is the first application of LC-MS/MS to field-collected arthropod disease vectors, the first use of LC-MS/MS in this disease system, and the first comparison of LC-MS/MS to DNA-based methods. We were able to detect blood meals in three insect vectors where nothing was detected using DNA-based methods and we showed the potential to quantify a blood meal using synthetic AQUA peptides. These results are important because understanding blood meal composition can lead to the development of Ecohealth control strategies for Chagas disease transmission, aiding in understanding and managing disease transmission dynamics.

### Verification with mouse blood

Our findings show LC-MS/MS to be a valuable tool in addition to DNA analysis for identification of blood meal sources in triatomine disease vectors. We used mouse blood to verify our ability to detect hemoglobin protein peptides with LC-MS/MS. We found the highest match to *M*. *musculus*; over 90% of the peptide positions along alpha and beta hemoglobin and over 90% of the spectra, matched *M*. *musculus*-reported sequences in GenBank. There is evidence that preservation methods influenced the number of peptides recovered from a blood sample. Twice as many peptide variants and more non-redundant peptides were recovered from both samples stored in EDTA.

Across the mouse blood samples, the only two peptides that did not match *M*. *musculus* hemoglobin entries currently in GenBank were alpha_17–31 and beta_18–30. All three control mouse blood samples identified both the expected version of alpha 17–31 (IGGHGAEYGAEALER) matching *M*. *musculus*, while one blood samples (2308) had an unexpected variant (VGSHAGEYGAEALER) currently reported in two species in the Ochotonidae (pika) family, *Tapirus terrestris* (Brazilian tapir), and *Microtus oeconomus* (root vole). An undescribed polymorphism in *M*. *musculus* is the most probable explanation for this non-match.

The second non-match peptide, beta_18–31 peptide NVADEVGGEALGR, matched another rodent, *Tamiasciurus hudsonicus*, the American red squirrel, and occurred in two mouse blood samples (2308, 2310). Although not detected with our current stringent filtering, the very similar peptide VNADEVGGEALGR differing by the inversion of the first two amino acids on the N-terminal end does match *M*. *musculus*. The detection of beta_18–31 depends on the presence and correct identification of b1 (N or V) and b2 (NV or VN) ions (and corresponding y ions) in the MS2 spectra. Generally, the lowest *m/z* b-type and highest *m/z* y-type ions are of lower abundance which could have led to the misidentification of this peptide. NVADEVGGEALGR (or VNADEVGGEALGR) is routinely observed with a relatively high signal. However, determination of the sequence at the extreme N-terminus is challenging. Since the blood source in this case is known, *M*. *musculus*, we infer that this peptide is indeed from mouse.

In spite of potential unreported polymorphisms or potential misattribution by the mass spectrometry search software, our results still support the top blood source match with high likelihood. When quantifying the percent match of the amino acid sequences of the peptides identified, 98% (2310) and 96% (sample 2308) of amino acids identified support *M*. *musculus* as the correct blood source. This is comparable to the DNA best match sequence identification (99–100%).

### Blood meal identification from field-collected insect vectors

With the *T*. *dimidiata* insect samples, we provide the first analysis of field-collected specimens with LC-MS/MS. Similar to the analysis of the mouse control samples, almost all of the peptides identified from the field-collected specimens matched a single blood source. Of the 22 non-redundant peptides identified from 50 spectral counts, only two peptides (2/50 spectral counts, 4%) had not been previously reported in the taxon with the strongest support. For sample FER051, beta_66–76 had previously been reported in two species in the squirrel (Sciuridae) family and differed by one amino acid from the fully tryptic peptides previously reported for cat, (VLDSFSDGLK in squirrel, VLNSFSDGLK in cat, Table 5). In addition to a polymorphism, this sequence could arise from a chemical conversion such as deamidation [60]. Overall, the percentage of amino acids identified that matched the top species, *F*. *catus* was 99.5%. For sample FER101, one peptide variant of alpha_62–90 was previously reported in *Leptonychotes weddellii* (Weddell seal) and also differed by only one amino acid from the peptide matching dog, the most highly supported blood source for that particular sample (VADALTTAVAHLDDLPGALSALSDLHAYK for dog, VADALTTAVSHIDDLPGALSALSDLHAYK for seal, Table 5). Of note, standard mass spectrometry analysis does not differentiate between leucine (L) and isoleucine (I) as these two amino acids have the same mass to charge ratio [61].

The amino acid difference for sample FER101 is in alpha hemoglobin site 71, one of the most variable hemoglobin amino acid positions in *Peromyscus* species [47]. Because there was only one protein sequence for alpha and beta hemoglobin from a domestic dog in GenBank when we made our database, an unreported variation in dog is the likely explanation for the difference. Polymorphisms in hemoglobin are known to expand along an altitudinal gradient [41, 47, 62] and are a more likely explanation than the insect vector having fed on seal at least 10,000 kilometers away in Antarctica. The villages in El Salvador where these samples were taken are in more rural areas, so a previously unknown polymorphism in the local dog population is a possibility. Again, 99.5% of the amino acid sequence of the peptides identified matched the top match blood meal source, *C*. *lupus*, supporting the high likelihood of the triatomine indeed having fed on domestic dog.

In spite of the fact that triatomine vectors digest blood meals slowly (up to 10 weeks) [14, 16, 24, 29], DNA-based methods for identifying blood meal sources fail in upwards of 50% of Chagas insect vectors [23, 24, 33–35, 37, 38, 63, 64]. In this regard, hemoglobin is a particularly stable protein and appears to last longer than DNA signatures [39]. Hemoglobin protein-based LC-MS/MS methods could be used when a sample does not show results with traditional DNA methods.

Interestingly, three of the four vectors took blood meals from dogs. Domestic dogs are considered the most important domestic reservoir for Chagas disease, although they may serve as a bridge species as well, connecting sylvatic and domestic cycles [65–67]. Previous studies have shown that *T*. *infestans* vectors are more likely to take a blood meal from a dog than a human [2, 68, 69], however keeping approximately two dogs in the household can increase human disease prevalence [2]. The results of our study of *T*. *dimidiata* from El Salvador supports previous empirical data that triatomine insect vectors feed on domestic dogs [65, 68–72]. One of the four vectors collected in the domestic environment within the house fed on cat. Cats as blood meal hosts are thought to occur much less frequently than dogs [2, 19, 73]. Still, cats can serve as both a blood meal for the vectors and host for *T*. *cruzi* [66, 68].

Finally, we demonstrate the potential of mass spectrometry-based methods to quantitatively profile triatomine blood meal sources. Using a stable isotope-labeled synthetic standard we show the quantification of a specific hemoglobin peptide derived from a field-collected sample (Fig 6). Using defined amounts of synthetic standards enables the absolute quantification of a given peptide in a sample. This could be a useful approach to quantify both absolute amounts of hemoglobin generally, as well as relative hemoglobin amounts from specific taxa when a vector has acquired blood meals from more than one species. For example, if a triatomine fed on both a dog and a human, there exist hemoglobin peptides that are common to the two mammals, as well as peptides that are unique to each. If 100 fmol of total hemoglobin was detected by measuring peptides in common between the two species, but species-specific peptides measured 30 fmol of dog peptides to 70 fmol of human peptide one could estimate the relative contribution of each to the blood meal derived from the vector. Additionally, if the sum of the two species-specific peptides fell short of the 100 fmol, conservation of mass would require the presence of a third blood meal, or at least an unanticipated polymorphism. The ability to quantify species-specific blood meals would help determine relative feeding prevalence of triatomines in an area with multiple potential hosts, and by extension would help focus management strategies based on particular host species.

Although LC-MS/MS presents a valuable tool in addition to DNA-based techniques, its cost could limit its application to situations where DNA analysis fails on a significant number of samples [74]. The upfront cost of LC-MS/MS platforms can be substantial, however the analysis itself can be relatively inexpensive given that these platforms typically exist in a multi-user facility with institutional support, as described previously [30] (S3 Table). In addition, LC-MS/MS can be accessible to researchers anywhere with such platforms being widely distributed, similarly to DNA sequencing facilities [30]. Costs per sample for a single LC-MS/MS run can range anywhere from $10 to $100 per sample or more when using multi-user facilities with proteomic platforms (S3 Table). Successful application of LC-MS/MS depends on a large and robust underlying database of hemoglobin protein sequences, just as DNA analysis depends on available DNA sequences. It is possible that a species would not be represented in the database and therefore not identified. However, the high conservation of hemoglobin would likely permit the identification of any blood meal to at least an evolutionarily-related species [30].

Our study detailing a protein-based mass spectrometry approach demonstrates proof-of principle support for identifying blood meal sources in Chagas disease insect vectors using hemoglobin peptides with surprisingly species-level resolution. Knowing how blood meals are changing in endemic areas is critical for vector control. Information such as blood meal prevalence leading to adjusting policies to manage potential animal hosts and insect vectors play increasingly important roles in controlling disease and preventing parasite infection, as vector control is still one of the most efficient control strategies [2, 14]. The experiments presented here show that using LC-MS/MS is a sensitive, valuable methodology to identify blood meal sources in Chagas disease triatomine insect vectors.

## Supporting information

S1 Fig Pipeline for inferring blood meal sources from hemoglobin peptides illustrated for mouse blood 1996. (PDF)

S2 Fig Pipeline for inferring blood meal sources from hemoglobin peptides illustrated for mouse blood 2308. (PDF)

S3 Fig Pipeline for inferring blood meal sources from hemoglobin peptides illustrated for mouse blood 2310. (PDF)

S4 Fig Pipeline for inferring blood meal sources from hemoglobin peptides illustrated for *T*. *dimidiata* insect vector sample 051. (PDF)

S5 Fig Pipeline for inferring blood meal sources from hemoglobin peptides illustrated for *T*. *dimidiata* insect vector sample 076. (PDF)

S6 Fig Pipeline for inferring blood meal sources from hemoglobin peptides illustrated for *T*. *dimidiata* insect vector sample 101. (PDF)

S7 Fig Pipeline for inferring blood meal sources from hemoglobin peptides illustrated for *T*. *dimidiata* insect vector sample 112. (PDF)

S1 Table Ion table providing the expected and observed *m/z* values for singly-charged fragment ions for peptide ion YFDSFGDLSSASAIMGNPK derived from spectrum shown in Fig 3C. Proline is underlined as it is the variable amino acid between the two peptides ions described in Fig 3C.
(PDF)

S2 Table Ion table providing the expected and observed *m/z* values for singly-charged fragment ions for peptide ion YFDSFGDLSSASAIMGNAK derived from the spectrum shown in Fig 3C. Alanine is underlined as it is the variable amino acid between the two peptides ions described in Fig 3C.
(PDF)

S3 Table Per sample cost of LC-MS/MS platform in four proteomics core facilities. Prices for the University of Vermont Proteomics Core Facility are for the high-resolution linear ion trap-orbitrap (LTQ-Orbitrap; Thermo Electron, Waltham, Massachusetts, USA) used for this study. Prices shown for other facilities are for comparable instruments. Self-run LC-MS/MS platforms can be run for as low as $4.75 per sample (see Önder et al. 2013, Supplementary Table S3 for further details).
(PDF)
