# Supplementary material for: Chagas disease vector blood meal sources identified by protein mass spectrometry
Source: PLoS One. 2017 Dec 12;12(12):e0189647. doi: 10.1371/journal.pone.0189647 (PMC5726658; doi:10.1371/journal.pone.0189647)
Supplement: S1 Fig — (PDF) [file pone.0189647.s001.pdf]

Sample: 1996 non-redundant peptides identified in sample

M. musculus NP_032244.2, BAG16710.1	alpha_17-31	alpha_32-40	alpha_41-56	alpha_91-99	alpha_128-139	beta_1-8	beta_18-30	beta_41-59		beta_66-82	beta_83-95	beta_105-120		beta_121-132	beta_133-144	beta_133-146	**Total**			
no. peptide variants	1	1	1	1	1	1	1	2		1	1	2		1	1	1	16			
spectral count	1	2	2	1	1	1	5	26	5	1	1	2	1	3	1	1	54			
**taxonomic affiliations**																	**range**			
no. of classes	1	1	1	3	1	1	1	1	1	1	1	1	1	1	1	1	(1- 3)			
no. of orders	1	1	2	50	8	2	1	1	1	1	3	1	1	2	7	6	(1 - 51)			
no. of families	6	2	7	127	27	4	1	1	1	1	6	1	1	4	16	15	(1 - 128)			
no. of genera	7	2	11	290	62	8	1	1	2	1	14	2	1	5	26	25	(1 - 291)			
no. of species	9	2	19	442	93	12	3	8	7	4	32	8	3	13	44	43	(2 - 443)			
**Species reported with peptide**																	**Total peptide matches per taxon**	**Total peptide non-matches per taxon**	**Percent peptides identified matching**	**Percent spectral count matching**
Mus musculus	x	x	x	x	x	x	x	x	x	x	x	x	x	x	x	x	16	0	100.00%	100.00%
Mus spicilegus							x	x	x	x	x	x	x	x	x	x	10	6	62.50%	85.20%
Mus macedonicus							x	x	x		x	x	x	x	x	x	9	7	56.30%	83.30%
Mus spretus								x		x	x	x		x			5	11	31.30%	61.10%
Cricetomys gambianus	x	x	x	x	x												5	11	31.30%	13.00%
Mus pahari										x					x	x	3	13	18.80%	5.60%
no. species not listed	5		15	434	88	9		4	4		28	4		7	38	37				
