# Supplementary material for: Chagas disease vector blood meal sources identified by protein mass spectrometry
Source: PLoS One. 2017 Dec 12;12(12):e0189647. doi: 10.1371/journal.pone.0189647 (PMC5726658; doi:10.1371/journal.pone.0189647)
Supplement: S2 Fig — (PDF) [file pone.0189647.s002.pdf]

Sample: 2308 non-redundant peptides identified in sample

M. musculus NP_032244.2, BAG16710.1	alpha_17-31		alpha_32-40	alpha_41-56	beta_18-30		beta_41-59		beta_66-82	beta_67-82	beta_67-76	beta_96-104	beta_105-120	Total			
no. peptide variants	2		1	1	2		2		1	1	1	1	1	13			
spectral count	2	1[a]	1	1	3	11[a]	24	3	2	5	1	1	1	56			
**taxanomic affiliations**														**range**			
no. of classes	1	1	1	1	1	1	1	1	1	1	1	3	1	(1- 3)			
no. of orders	1	3	1	2	1	1	1	1	1	1	1	55	1	(1 - 55)			
no. of families	6	3	2	7	1	1	1	1	1	1	1	126	1	(1 - 126)			
no. of genera	7	3	2	11	1	1	1	2	1	1	1	262	2	(1 - 262)			
no. of species	9	4	2	19	3	1	8	7	4	4	4	392	8	(1-392)			
**Species reported with peptide**														**Total peptide matches per taxon**	**Total peptide non-matches per taxon**	**Percent peptides identified matching**	**Percent spectral count matching**
Mus musculus	x		x	x	x		x	x	x	x	x	x	x	11	2	84.6%	78.57%
Mus spicilegus					x		x	x	x	x	x	x	x	8	5	61.5%	71.43%
Mus macedonicus					x		x	x			x	x	x	6	7	46.2%	58.93%
Mus spretus							x		x	x		x	x	5	8	38.5%	58.93%
Mus saxicola							x	x			x	x	x	5	8	38.5%	53.57%
Mus pahari									x	x		x		3	10	23.1%	14.29%
Cricetomys gambianus	x		x	x										3	10	23.1%	7.14%
Nannospalax ehrenbergi	x			x										2	11	15.4%	5.36%
Nannospalax galili	x			x										2	11	15.4%	5.36%
Tamiasciurus hudsonicus						x						x		2	11	15.4%	21.43%
Ochotona erythrotis		x		x										2	11	15.4%	3.57%
Ochotona dauurica		x		x										2	11	15.4%	3.57%
Tapirus terrestris		x										x		2	11	15.4%	3.57%
Microtus oeconomus		x		x										2	11	15.4%	3.57%
no. species not listed	5			12			3	3				384	3				

[a] indicates peptides not matching the known blood sources, *M. musculus*
