# Supplementary material for: Chagas disease vector blood meal sources identified by protein mass spectrometry
Source: PLoS One. 2017 Dec 12;12(12):e0189647. doi: 10.1371/journal.pone.0189647 (PMC5726658; doi:10.1371/journal.pone.0189647)
Supplement: S3 Fig — (PDF) [file pone.0189647.s003.pdf]

Sample: 2310

M. musculus NP_032244.2, BAG16710.1	alpha_17-31	alpha_62-90	beta_9-17	beta_18-30	beta_41-59	beta_121-132	beta_133-144	Total			
	non-redundant peptides identified in sample										
no. peptide variants	1	1	1	1	1	1	1	7			
spectral count	1	5	6	11[a]	27	1	4	55			
**taxanomic affiliations**								**range**			
no. of classes	1	1	1	1	1	1	1	(1)			
no. of orders	1	1	2	1	1	2	2	(1 - 2)			
no. of families	6	2	3	1	1	4	2	(1 - 6)			
no. of genera	7	2	3	1	1	5	2	(1 - 7)			
no. of species	9	3	10	1	8	13	4	(1-13)			
**Species reported with peptide**								**Total peptide matches per taxon**	**Total peptide non-matches per taxon**	**Percent peptides identified matching**	**Percent spectral count matching**
Mus musculus	x	x	x		x	x	x	6	1	85.7%	80.00%
Mus spretus			x		x	x	x	4	3	57.1%	69.09%
Nannospalax galili	x	x	x			x		4	3	57.1%	23.64%
Mus cervicolor			x		x		x	3	4	42.9%	67.27%
Nannospalax ehrenbergi	x	x				x		3	4	42.9%	12.73%
Tamiasciurus hudsonicus				x				1	6	14.3%	0.00%
Tarsius syrichta							x	1	6	14.3%	7.27%
no. species not listed	6		6		5	9					

[a] indicates peptides not matching the known blood sources, *M. musculus*
