# Supplementary material for: Chagas disease vector blood meal sources identified by protein mass spectrometry
Source: PLoS One. 2017 Dec 12;12(12):e0189647. doi: 10.1371/journal.pone.0189647 (PMC5726658; doi:10.1371/journal.pone.0189647)
Supplement: S4 Fig — (PDF) [file pone.0189647.s004.pdf]

Sample: FER 051 non-redundant peptides identified in sample

F.catus P07405.1, XP_003992931.2	alpha_41-56	alpha_62-90	beta_18-30	beta_41-59	beta_66-76	beta_67-76	beta_133-144	**Total**			
no. peptide variants	1	1	1	1	1	1	1	7			
spectral count	1	4	1	3	1	2	1	13			
**taxanomic affiliations**								**range**			
no. of classes	2	1	1	1	1	1	1	(1- 3)			
no. of orders	7	1	4	1	1	4	1	(1 - 51)			
no. of families	26	1	25	1	1	13	3	(1 - 128)			
no. of genera	61	1	62	1	2	21	7	(1 - 291)			
no. of species	94	1	88	1	2	26	10	(2 - 443)			
**Species reported with peptide**								**Total peptide matches per taxon**	**Total peptide non-matches per taxon**	**Percent peptides identified matching**	**Percent spectral count matching**
Felis catus	x	x	x	x		x	x	6	1	85.7%	92.31%
Sciurus carolinensis					x			1	6	14.3%	7.69%
Tamias striatus					x			1	6	14.3%	7.69%
no. species not listed	93		87			25	9				
