# Supplementary material for: Chagas disease vector blood meal sources identified by protein mass spectrometry
Source: PLoS One. 2017 Dec 12;12(12):e0189647. doi: 10.1371/journal.pone.0189647 (PMC5726658; doi:10.1371/journal.pone.0189647)
Supplement: S5 Fig — (PDF) [file pone.0189647.s005.pdf]

**Sample: FER 076** non-redundant peptides identified in sample

| *C. lupus* *P60529.1, P60524.1* | beta_9-17 | beta_18-30 | beta_31-40 | **Total** |
|---|---|---|---|---|
| no. amino acids/peptide | 9 | 13 | 10 | 32 |
| no. peptide variants | 1 | 1 | 1 | 3 |
| spectral count | 1 | 1 | 1 | 3 |
| **taxanomic affiliations** | | | | **range** |
| no. of classes | 1 | 1 | 4 | (1- 3) |
| no. of orders | 1 | 4 | 42 | (1 - 51) |
| no. of families | 1 | 25 | 79 | (1 - 128) |
| no. of genera | 2 | 62 | 167 | (1 - 291) |
| no. of species | 3 | 88 | 242 | (2 - 443) |

| **Species reported with peptide** | | | | **Total peptide matches per taxon** | **Total peptide non-matches per taxon** | **Percent peptides identified matching** | **Percent spectral count matching** |
|---|---|---|---|---|---|---|---|
| *Canis lupus* | x | x | x | 3 | 0 | 100.0% | 100.00% |
| *Canis latrans* | x | x | x | 3 | 0 | 100.0% | 100.00% |
| *Chrysocyon brachyurus* | x | x | x | 3 | 0 | 100.0% | 100.00% |
| no. species not listed | | 82 | 237 | | | | |
