# Supplementary material for: Chagas disease vector blood meal sources identified by protein mass spectrometry
Source: PLoS One. 2017 Dec 12;12(12):e0189647. doi: 10.1371/journal.pone.0189647 (PMC5726658; doi:10.1371/journal.pone.0189647)
Supplement: S6 Fig — (PDF) [file pone.0189647.s006.pdf]

Sample: FER 101 non-redundant peptides identified in sample

C. lupus *P60529.1, P60524.1*	alpha_17-31	alpha_32-40	alpha_41-56	alpha_61-90	alpha_62-90		alpha_91-99	alpha_128-139	beta_1-8	beta_18-30	beta_41-59	beta_96-104	beta_133-144	**Total**			
no. amino acids/peptide	15	9	16	30	29	29	9	12	8	13	19	9	12	210			
no. peptide variants	1	1	1	1	2		1	1	1	1	1	1	1	13			
spectral count	1	1	3	1	2	1[a]	1	3	3	1	3	2	1	23			
**taxanomic affiliations**														**range**			
no. of classes	1	1	2	1	1	1	3	1	1	1	1	4	1	(1- 3)			
no. of orders	1	1	8	1	1	1	50	2	7	4	1	21	14	(1 - 51)			
no. of families	1	1	15	1	1	1	127	2	10	25	1	51	45	(1 - 128)			
no. of genera	4	4	36	4	4	1	290	4	15	62	1	97	92	(1 - 291)			
no. of species	5	5	41	5	5	1	442	4	20	88	3	144	125	(2 - 443)			
**Species reported with peptide**														**Total peptide matches per taxon**	**Total peptide non-matches per taxon**	**Percent peptides identified matching**	**Percent spectral count matching**
Canis lupus	x	x	x	x	x		x	x	x	x	x	x	x	12	1	92.3%	95.65%
Canis latrans	x	x	x	x	x		x		x	x	x	x	x	11	2	84.6%	82.61%
Chrysocyon brachyurus	x	x	x	x	x		x		x	x	x	x	x	11	2	84.6%	92.31%
Cerdocyon thous	x	x	x	x	x		x		x	x		x	x	10	3	76.9%	69.57%
Vulpes vulpes	x	x	x	x	x		x		x	x		x	x	10	3	76.9%	69.57%
Leptonychotes weddellii			x			x	x		x	x		x	x	7	6	53.8%	52.17%
Eulemur fulvus							x	x				x	x	4	9	30.8%	30.43%
Hapalemur griseus							x	x						2	11	15.4%	17.39%
Varecia variegata							x	x						2	11	15.4%	17.39%
no. species not listed			35				433		14	82		137	118				

[a] indicates peptides not matching top match blood source, *C. lupus*
