# Supplementary material for: Chagas disease vector blood meal sources identified by protein mass spectrometry
Source: PLoS One. 2017 Dec 12;12(12):e0189647. doi: 10.1371/journal.pone.0189647 (PMC5726658; doi:10.1371/journal.pone.0189647)
Supplement: S7 Fig — (PDF) [file pone.0189647.s007.pdf]

Sample: FER 112 non-redundant peptides identified in sample

C. lupus *P60529.1, P60524.1*	alpha_17-31	alpha_32-40	alpha_62-90	alpha_128-139	beta_31-40	beta_41-59	beta_67-76	beta_121-132	beta_133-144	Total			
no. amino acids/peptide	15	9	29	12	10	19	10	12	12	128			
no. peptide variants	1	1	1	1	1	1	1	1	1	9			
spectral count	3	2	2	1	2	3	1	2	1	17			
**taxanomic affiliations**										**range**			
no. of classes	1	1	1	1	4	1	1	1	1	(1- 3)			
no. of orders	1	1	1	2	42	1	4	4	14	(1 - 51)			
no. of families	1	1	1	2	79	1	13	23	45	(1 - 128)			
no. of genera	4	4	4	4	167	1	21	55	92	(1 - 291)			
no. of species	5	5	5	4	242	3	26	79	125	(2 - 443)			
**Species reported with peptide**										**Total peptide matches per taxon**	**Total peptide non-matches per taxon**	**Percent peptides identified matching**	**Percent spectral count matching**
Canis lupus	x	x	x	x	x	x	x	x	x	9	0	100.0%	100.00%
Canis latrans	x	x	x		x	x	x	x	x	8	1	88.9%	94.12%
Chrysocyon brachyurus	x	x	x		x	x	x	x	x	8	1	88.9%	94.12%
Cerdocyon thous	x	x	x		x		x	x	x	7	2	77.8%	64.71%
Vulpes vulpes	x	x	x		x		x	x	x	7	2	77.8%	76.47%
Eulemur fulvus				x					x	2	7	22.2%	11.76%
Hapalemur griseus				x						1	8	11.1%	5.88%
Varecia variegata				x						1	8	11.1%	5.88%
no. species not listed					237		21	74	119				
