# Supplementary material for: Chagas disease vector blood meal sources identified by protein mass spectrometry
Source: PLoS One. 2017 Dec 12;12(12):e0189647. doi: 10.1371/journal.pone.0189647 (PMC5726658; doi:10.1371/journal.pone.0189647)
Supplement: S1 Table — Proline is underlined as it is the variable amino acid between the two peptides ions described in Fig 3C. (PDF) [file pone.0189647.s008.pdf]

Sequence	#	b-ions *m/z* values expected	b-ions *m/z* values observed	y-ions *m/z* values expected	y-ions *m/z* values observed	#
Y	1	164.07065	-	2006.91646	-	19
F	2	311.13906	311.141	1843.85313	-	18
D	3	426.16601	426.166	1696.78472	1696.782	17
S	4	513.19803	513.198	1581.75778	1581.758	16
F	5	660.26645	660.266	1494.72575	1494.726	15
G	6	717.28791	717.289	1347.65733	1347.658	14
D	7	832.31485	832.315	1290.63587	1290.637	13
L	8	945.39892	945.399	1175.60893	1175.609	12
S	9	1032.43095	1032.462	1062.52486	1062.525	11
S	10	1119.46297	1119.462	975.49284	975.493	10
A	11	1190.50009	1190.300	888.46081	888.461	9
S	12	1277.53212	1277.532	817.42369	817.424	8
A	13	1348.56923	1348.569	730.39167	730.391	7
I	14	1461.65329	1461.654	659.35455	659.354	6
M	15	1592.69378	1592.695	546.27049	546.270	5
G	16	1649.71524	1649.717	415.23000	415.230	4
N	17	1763.75817	1763.760	358.20854	358.208	3
P	18	1860.81093	-	244.16561	-	2
K	19	1988.90590	-	147.11285	-	1
