# Supplementary material for: Chagas disease vector blood meal sources identified by protein mass spectrometry
Source: PLoS One. 2017 Dec 12;12(12):e0189647. doi: 10.1371/journal.pone.0189647 (PMC5726658; doi:10.1371/journal.pone.0189647)
Supplement: S2 Table — Alanine is underlined as it is the variable amino acid between the two peptides ions described in Fig 3C. (PDF) [file pone.0189647.s009.pdf]

**Sequence**	**#**	**b-ions *m/z* values**		**y-ions *m/z* values**		**#**
		expected	observed	expected	observed	
Y	1	164.07065	-	1980.90081	-	19
F	2	311.13906	-	1817.83748	-	18
D	3	426.16601	426.166	1670.76907	-	17
S	4	513.19803	513.197	1555.74213	1555.742	16
F	5	660.26645	660.266	1468.71010	1468.712	15
G	6	717.28791	717.285	1321.64168	1321.642	14
D	7	832.31485	832.315	1264.62022	1264.621	13
L	8	945.39892	945.399	1149.59328	1149.593	12
S	9	1032.43095	1032.431	1036.50921	1036.509	11
S	10	1119.46297	1119.465	949.47719	949.477	10
A	11	1190.50009	1190.500	862.44516	862.445	9
S	12	1277.53212	1277.532	791.40804	791.408	8
A	13	1348.56923	1348.568	704.37602	704.375	7
I	14	1461.65329	1461.654	633.33890	633.338	6
M	15	1592.69378	1592.695	520.25484	520.254	5
G	16	1649.71524	1649.712	389.21435	389.214	4
N	17	1763.75817	1763.753	332.19289	-	3
A	18	1834.79528	1834.796	218.14996	-	2
K	19	1962.89025	-	147.11285	-	1
