# Supplementary material for: Chagas disease vector blood meal sources identified by protein mass spectrometry
Source: PLoS One. 2017 Dec 12;12(12):e0189647. doi: 10.1371/journal.pone.0189647 (PMC5726658; doi:10.1371/journal.pone.0189647)
Supplement: S3 Table — Prices for the University of Vermont Proteomics Core Facility are for the high-resolution linear ion trap-orbitrap (LTQ-Orbitrap; Thermo Electron, Waltham, Massachusetts, USA) used for this study. Prices shown for other facilities are for comparable instruments. Self-run LC-MS/MS platforms can be run for as low as $4.75 per sample (see Önder et al. 2013, Supplementary Table S3 for further details). (PDF) [file pone.0189647.s010.pdf]

Facility	Per sample cost of LC-MS/MS run	
	In-house[a]	Academic[b]
Harvard Mass Spectrometry and Proteomics Resource Laboratory	$100	$160
University of California Davis Proteomics Core Facility	$73	$114
Nebraska Center for Biotechnology Proteomics and Metabolomics Facility	$40	$90
University of Vermont Proteomics Core Facility	$10	$10[c]

[a] in-house pricing for investigators that have access to proteomic core facilities; [b] academic pricing for samples from outside the listed proteomic core facility; [c] UVM works with outside investigators on a collaborative basis with an expectation of shared authorship in publications
